# Developmental transcriptome analysis and identification of genes involved in formation of intestinal air-breathing function of Dojo loach, *Misgurnus anguillicaudatus*

**DOI:** 10.1038/srep31845

**Published:** 2016-08-22

**Authors:** Weiwei Luo, Xiaojuan Cao, Xiuwen Xu, Songqian Huang, Chuanshu Liu, Tea Tomljanovic

**Affiliations:** 1 College of Fisheries, Key Lab of Agricultural Animal Genetics, Breeding and Reproduction of Ministry of Education/Key Lab of Freshwater Animal Breeding, Ministry of Agriculture, Huazhong Agricultural University, Wuhan 437000, Hubei, People’s Republic of China; 2 Freshwater Aquaculture Collaborative Innovation Center of Hubei Province, Hubei, People’s Republic of China; 3 Department for Fisheries, Beekeeping, Game management and Special Zoology, Faculty of Agriculture, University of Zagreb, Zagreb, Croatia

## Abstract

Dojo loach, *Misgurnus anguillicaudatus* is a freshwater fish species of the loach family Cobitidae, using its posterior intestine as an accessory air-breathing organ. Little is known about the molecular regulatory mechanisms in the formation of intestinal air-breathing function of *M. anguillicaudatus*. Here high-throughput sequencing of mRNAs was performed from six developmental stages of posterior intestine of *M. anguillicaudatus*: 4-Dph (days post hatch) group, 8-Dph group, 12-Dph group, 20-Dph group, 40-Dph group and Oyd (one-year-old) group. These six libraries were assembled into 81300 unigenes. Totally 40757 unigenes were annotated. Subsequently, 35291 differentially expressed genes (DEGs) were scanned among different developmental stages and clustered into 20 gene expression profiles. Finally, 15 key pathways and 25 key genes were mined, providing potential targets for candidate gene selection involved in formation of intestinal air-breathing function in *M. anguillicaudatus*. This is the first report of developmental transcriptome of posterior intestine in *M. anguillicaudatus*, offering a substantial contribution to the sequence resources for this species and providing a deep insight into the formation mechanism of its intestinal air-breathing function. This report demonstrates that *M. anguillicaudatus* is a good model for studies to identify and characterize the molecular basis of accessory air-breathing organ development in fish.

Dojo loach, *Misgurnus anguillicaudatus*, is a freshwater fish species of the loach family Cobitidae^1^. The species is native to East Asia, but is also popular as an aquarium fish and has been introduced elsewhere in Asia and to Europe and North America^2^. The loach inhabits mud, ponds, and rice fields, which are subject to periodic drying^3^. During drying, loaches burrow under the mud. In such poor dissolved oxygen conditions, their respiratory system has been the focus of much interest. The loaches have three forms of respiration: branchial, cutaneous and intestinal. The first two types are common to other fish and amphibian. Intestinal respiration, however, is found in Cobitidae^4^. The intestine of loaches can be divided into anterior, mid, and posterior sections. The anterior intestine and middle intestine are adapted to serve major role of digestion and absorption. The posterior intestine is highly modified, being well vascularized with intraepithelial capillaries, which makes it well suited for gas exchange^5^. Studies of intestinal air-breathing of the loach have focused on histological description of the intestine and physiological aspect of gas exchange^1^,^3^. Little is known about the molecular regulatory mechanisms in occurrence and formation of intestinal air-breathing function of *M. anguillicaudatus*.

With the advantage of low cost and high speed, massively parallel sequencing (Illumina) RNA-Seq analysis is now the most convenient method to find out new genes and investigate gene expression patterns of non-model organisms, especially for species of which the whole genome sequence is not yet available^6^. To date, several studies have reported on the gene expression profiles of different intestine tissues of fishes like Atlantic cod^7^, tilapia^8^ and zebrafish^9^. However, studies about the dynamic expression profiles on the intestine of *M. anguillicaudatus* remain scarce. Here we used high-throughput sequencing of mRNAs from posterior intestine of loaches at different development stages to research the genes involved in formation of intestinal air-breathing function of *M. anguillicaudatus*. We constructed six libraries including 4-Dph (days post hatch) group, 8-Dph group, 12-Dph group, 20-Dph group, 40-Dph group and Oyd (one year old) group. Hence, the major objective of this study was to investigate genes related to the formation of intestinal air-breathing function of *M. anguillicaudatus* using high-throughput sequencing and bioinformatic analysis.

## Results

### Histological observations of intestine tissues in *M. anguillicaudatus* during different developmental stages

**Figure 1:**
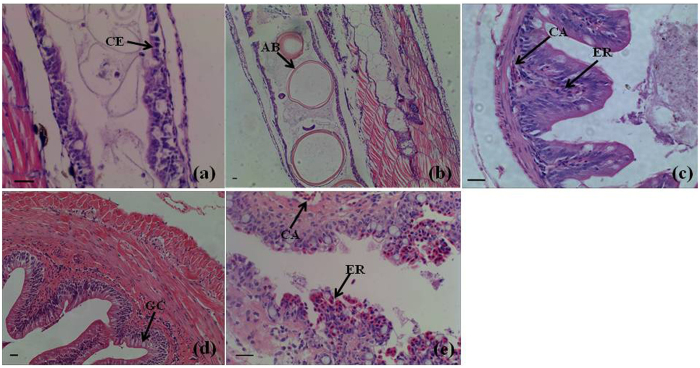
Histological observations of intestine tissues of *M. anguillicaudatus* during different developmental stages. (**a**) Longitudinal section of intestine from 8-Dph loach. (**b**) Longitudinal section of intestine from 10-Dph loach. (**c**) Cross section of posterior intestine from 40-Dph loach. (**d**) Cross section of anterior intestine from Oyd. (**e**) Cross section of posterior intestine from Oyd. Abbreviations: air bubble (AB); capillary vessel (CA); simple columnar epithelium (CE); erythrocyte (ER); goblet cell (GC). Bar indicated 20 μm.

The newly hatched loach larva had a long straight intestinal tube with a very simple structure. In order to uncover the relationship between the occurrence and formation of intestinal air-breathing function and the changes of intestinal structure, microstructures of intestine tissues in loach during different developmental stages were observed by using Delafield’s haematoxylin, counterstained with eosin (HE) staining. Intestinal epithelium of 8-Dph loach was simple columnar epithelium (Fig. 1a). Intestinal lumen of 10-Dph loach showed numerous air bubbles (Fig. 1b). The numerous erythrocytes and capillary vessels were showed in posterior intestine of 40-Dph loach (Fig. 1c). Histological observations of posterior intestines suggested the initial formation period of intestinal air-breathing function of loach began at around 10 days post hatch. With the development of posterior intestine of loach, especially adults, the obvious structural differences between anterior intestine and posterior intestine were present (Fig. 1d,e). Mucosal folds of anterior intestine of Oyd loach exhibited a more complicated, interconnecting arrangement, and were more extensive than those in the posterior intestine of Oyd loach. The anterior intestine of Oyd loach possessed the typical characteristics of an absorptive intestinal epithelium composed of columnar epithelial cells with abundant goblet cells and a brush border (Fig. 1d). While, the typical characteristics of posterior intestine of Oyd loach was abundant in blood capillaries within the mucosal epithelium, which made it well suited for gas exchange (Fig. 1e).

### Transcriptome sequencing and *de novo* assembly

Approximately 38.35 million, 40.18 million, 36.59 million, 37.79 million, 32.89 million and 58.15 million raw reads were sequenced using RNA-Seq technique in 4-Dph, 8-Dph, 12-Dph, 20-Dph, 40-Dph and Oyd libraries, respectively (Table 1). These raw reads were further filtered by removing adaptors, ambiguous nucleotides and low-quality sequences to generate clean reads. After trimming the raw reads, there were 37.79 million, 39.66 million, 36.03 million, 37.28 million, 32.45 million and 48.37 million clean reads generated from 4-Dph, 8-Dph, 12-Dph, 20-Dph, 40-Dph and Oyd, respectively (Table 1).

The clean reads obtained from the six different transcriptome libraries were pooled and assembled full-length transcripts without reference genomes by Trinity software. After the elimination of redundant transcripts, 81300 unigenes were achieved with an average length of 879.78 base pairs and N50 of 1662 bp (Supplementary Table S1). The size distribution of the unigenes is shown in Supplementary Fig. S1. The all unigenes provided a sequence basis for analysis of gene expression in posterior intestine of loach during different developmental stages.

### Unigene annotation to public databases

In order to maximize the information of assembled unigenes, all of the 81300 unigene sequences were searched against four public databases: NCBI non-redundant protein (Nr) database, Swiss-Prot protein database (Swissprot), euKaryotic Ortholog Groups (KOG) and Kyoto Encyclopedia of Genes and Genomes (KEGG) database. Among these unigenes, 40563 (49.89%), 31588 (38.85%), 24837 (30.55%), 17077 (21.00%) of the unigenes were identified in the Nr, Swissprot, KOG and KEGG database, respectively (Supplementary Table S2). Totally 40757 unigenes (50.13% of all the 81300 unigene sequences) were annotated in the four public databases. Among all the 40757 annotated unigenes, 14102 (27.98%) were simultaneously annotated by the four databases, and 32747 (80.35%) showed homology to the known sequences deposited in at least one database (Supplementary Fig. S2).

### Functional annotation and classification of unigenes

**Figure 2:**
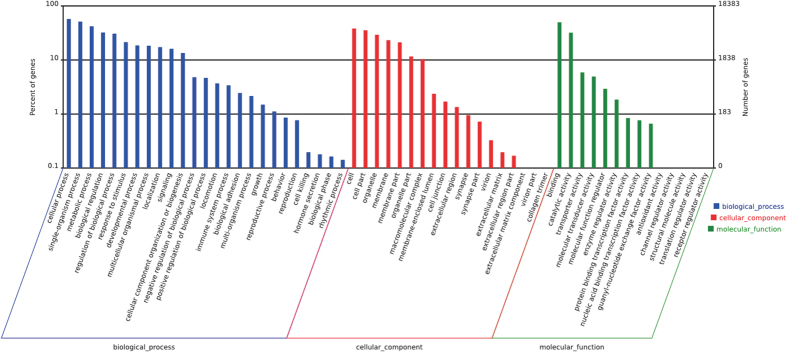
GO classifications of all unigenes from posterior intestine of *M. anguillicaudatus*.

GO assignment was performed to classify functions of the predicted loach genes. Based on sequence homology, annotated unigenes were classified into three major functional categories: biological process, cellular component and molecular function (Fig. 2). Under the biological process category, the major GO terms were cellular process (10512 unigenes), single-organism process (9414 unigenes), metabolic process (7709 unigenes), biological regulation (5919 unigenes), regulation of biological process (5634 unigenes), response to stimulus (3938 unigenes), developmental process (3417 unigenes) and multicellular organismal process (3365 unigenes). Within the cellular component category, a significant percentage of genes were clustered into cell (7023 unigenes), cell part (6520 unigenes), organelle (5358 unigenes), membrane (4274 unigenes) and membrane part (3892 unigenes). In the molecular function category, most genes were assigned to binding (9144 unigenes) and catalytic activity (5869 unigenes) followed by transporter activity (1081 unigenes), molecular transducer activity (907 unigenes) and molecular function regulator (537 unigenes).

**Figure 3:**
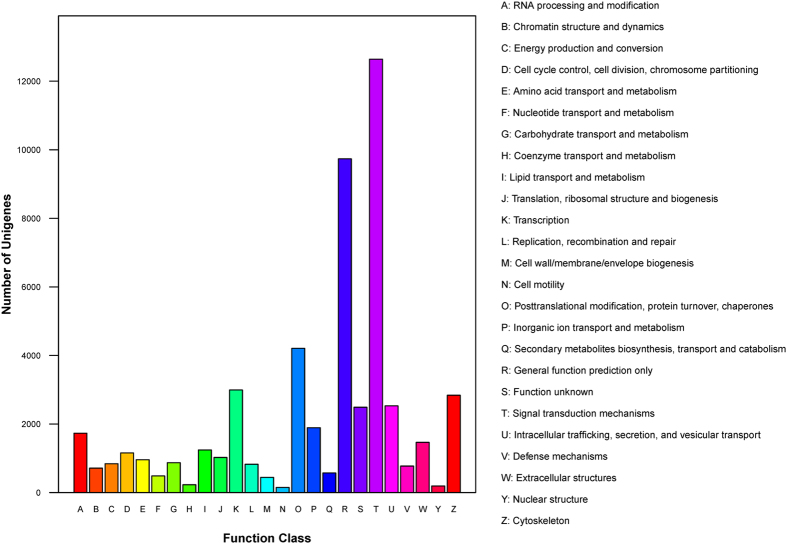
KOG classifications of all unigenes from posterior intestine of *M. anguillicaudatus*.

To classify orthologous proteins, the assembled unigenes were compared against KOG. 24837 unigenes were clustered into 25 categories (Fig. 3). Among them, the cluster for signal transduction mechanisms represented the largest group (12641 unigenes, 50.90%), followed by general function prediction only (9738 unigenes, 39.21%), posttranslational modification, protein turnover, chaperones (4209 unigenes, 16.95%), transcription (2993 unigenes, 12.05%), cytoskeleton (2841 unigenes, 11.44%), intracellular trafficking, secretion, vesicular transport (2530 unigenes, 10.19%), function unknown (2491 unigenes, 10.03%), inorganic ion transport and metabolism (1893 unigenes, 7.62%), RNA processing and modification (1732 unigenes, 6.97%) and extracellular structures (1466 unigenes, 5.90%).

KEGG pathway-based analyses help to identify the biological pathways that are related to unigenes. Totally, 17077 (41.90%) out of 40757 were assigned 240 KEGG pathways. Among these unigenes, top ten enrichment of KEGG pathway were metabolic pathways (ko01100, 1799 unigenes), pathways in cancer (ko05200, 763unigenes), regulation of actin cytoskeleton (ko04810, 622 unigenes), neuroactive ligand-receptor interaction (ko04080, 616 unigenes), MAPK signaling pathway (ko04010, 616 unigenes), dilated cardiomyopathy (ko05414, 582 unigenes), hypertrophic cardiomyopathy (HCM) (ko05410, 563 unigenes), focal adhesion (ko04510, 546 unigenes) and calcium signaling pathway (ko04020, 524 unigenes).

### Identification and functional analysis of differentially expressed genes

The DEGs during the course of posterior intestine development were explored by using DEGseq with the criteria of |log_2_Ratio| ≥ 1 and FDR ≤ 0.01. In total, the number of DEGs between two consecutive stages were as follows: 10279 between stages Dph-4 and Dph-8 (23.94% up- and 76.06% down-regulated in stage Dph-8), 9875 between stages Dph-8 and Dph-12 (74.10% up- and 25.90% down-regulated in stage Dph-12), 7107 between stages Dph-12 and Dph-20 (71.34% up- and 28.66% down-regulated in stage Dph-20), 11130 between stages Dph-20 and Dph-40 (69.20% up- and 30.80% down-regulated in stage Dph-40), and 21461 between stages Dph-40 and Oyd (53.04% up- and 46.96% down-regulated in stage Oyd). Details on the number of DEGs at each two consecutive time points are presented in Supplementary Table S3.

To better understand the dynamic changes of gene expression in posterior intestine during all the six developmental stages, further analyses of the DEGs were also performed between the two stages of Dph-4-VS-Dph-8, Dph-4-VS-Dph-12, Dph-4-VS-Dph-20, Dph-4-VS-Dph-40 and Dph-4-VS-Oyd, respectively (Supplementary Fig. S3). Finally, a total of 35291 genes were identified as DEGs among the six developmental stages.

To investigate the biological events that the DEGs mainly involved during posterior intestine development, GO term enrichment analysis were conducted (Supplementary Fig. S4). Under the biological process category, the major GO terms were cellular process (5603 unigenes), single-organism process (5064 unigenes), metabolic process (4190 unigenes), biological regulation (3175 unigenes), regulation of biological process (3021 unigenes), response to stimulus (2122 unigenes), developmental process (1862 unigenes) and multicellular organismal process (1810 unigenes). In the cellular component category, a significant percentage of genes were clustered into cell (3700 unigenes), cell part (3424 unigenes), organelle (2817 unigenes), membrane (2132 unigenes) and membrane part (1937 unigenes). Within the molecular function category, most genes were assigned to binding (4863 unigenes) and catalytic activity (3261 unigenes), followed by transporter activity (483 unigenes), molecular transducer activity (453 unigenes) and molecular function regulator (304 unigenes). These suggested that gene regulation was crucial for achieving various physiological functions associated to the development of loach posterior intestine.

KEGG pathway annotation enabled us to assign 35291 DEGs to 238 pathways. Supplementary Fig. S5 showed the top 20 significantly enriched KO pathways. The result revealed significant enrichment for 20 pathways with 17 metabolism pathways, two genetic information processing pathways and one organismal system pathway. These observations disclosed the vital implications of amino acid metabolism, lipid metabolism, metabolism of cofactors and vitamins, genetic information processing and organismal system in developmental processes of loach posterior intestine.

### Dynamic expression profiles of DEGs

**Figure 4:**
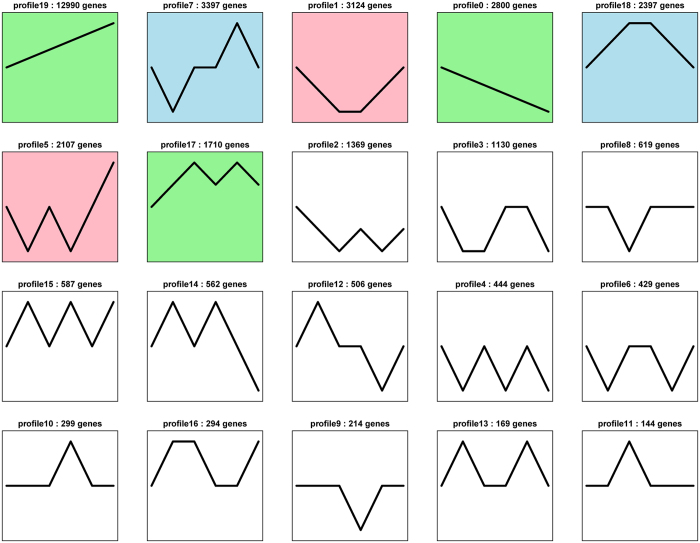
Expression profiles and clusters of DEGs obtained from the STEM clustering. Numbers indicated profiles or gene numbers. Significantly different profiles were represented by different background colors.

To get dynamic expression patterns of DEGs during development, Short Time-series Expression Miner (STEM) software was performed to classify all the DEGs according to their abundance changes. The 35291 DEGs were classified into 20 clusters according to their expression patterns (Fig. 4). Seven significant expression profiles (profile 0, profile 1, profile 5, profile 7, profile 17, profile 18 and profile 19) were identified. As shown in Fig. 4, significantly different profiles were represented by different background colors. Respectively, 2800, 3124, 2107, 3397, 1710, 2397 and 12990 co-expressed DEGs were identified for profile 0, profile 1, profile 5, profile 7, profile 17, profile 18 and profile 19. Of these, the most abundant group was profile 19, with 12990 genes whose expression showed a positive slope, which was reversed to the trend in profile 0. Interestingly, reversed trends were also observed between profile 1 and profile 18, profile 5 and profile 17, respectively. In order to obtain a glimpse of the biological functions of these 7 clusters of genes, GO enrichment analysis was conducted. The genes co-expressed in profile 0, profile 1, profile 5, profile 7, profile 17, profile 18 and profile 19, correspondingly participated in 1470, 1897, 1222, 760, 386, 801 and 2368 biological functions (GO terms).

Profile 18 and profile 19 were the two represented patterns. Profile 18 firstly increased, and reached to top in Dph-12 and Dph-20, and then decreased. It was consistent with the trend of occurred and formed process of intestinal air-breathing function in the loach. The GO functional analysis of profile 18 showed: under the biological process category, the significantly representatives included anatomical structure development, single-organism developmental process, anatomical structure morphogenesis, single-organism cellular process and multiple processes related to development; Within the cellular component category, a significant percentage of genes were clustered into cell and cell part; In the molecular function category, most genes were assigned to binding, catalytic activity and ion binding. The expression levels of DEGs of profile 19 was constantly increased across posterior intestine development stages, suggesting more and more genes were involved in the occurred and formed process of intestinal air-breathing function. The GO functional analysis of profile 19 showed: in the biological process category, cellular process, single-organism process, biological regulation, regulation of biological process and single-organism cellular process represented the majorities of this category; among cellular component terms, cell and cell part were observed to be the most abundant classes; binding, catalytic activity and molecular transducer activity showed a high percentage of genes in the category of molecular function. It was considered that DEGs of profile 18 and profile 19 were closely involved in formation of intestinal air-breathing function of the loach.

### Individual key pathways and DEGs related to formation of loach intestinal air-breathing function

**Figure 5:**
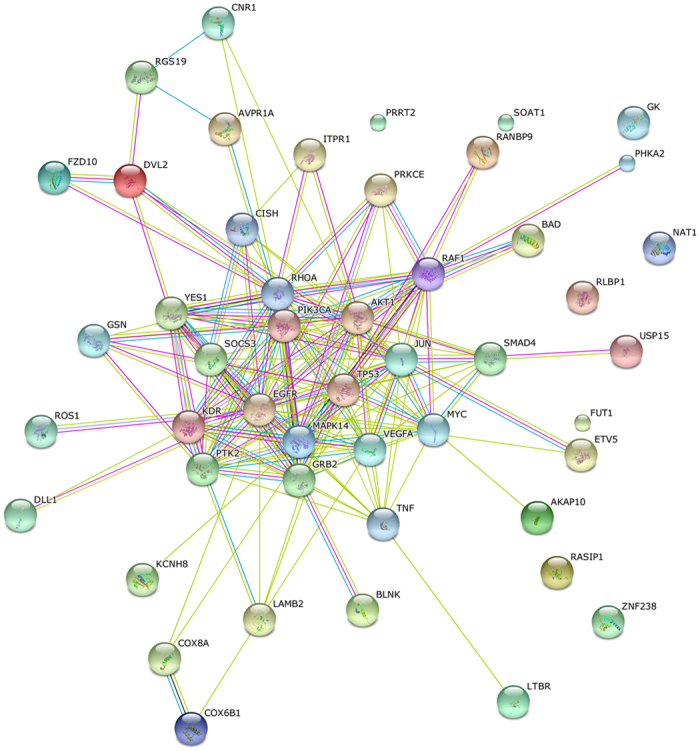
Protein-protein interaction network for the 50 important enzymes encoded by key DEGs of key pathways. Different nodes represented different enzymes. The interactions among these enzymes were represented by different colorful lines.

Fifteen key pathways (Table 2) and twenty-five key DEGs associated with the formation of intestinal air-breathing function in the loach were identified from profile 18 and profile 19. These key pathways were mainly involved in development, angiogenesis and cytoskeleton. Fifty important enzymes of the 15 key pathways were selected by three strategies simultaneously which were considered to be related to the occurred and formed process of intestinal air-breathing function: (i) 25 important enzymes that showed high participation frequency in the 15 key pathways of profile 18 and profile 19; (ii) another 20 important enzymes which appeared in some individual key pathways (10/15 pathways) of both profile 18 and profile 19; (iii) 5 important enzymes which showed in the other key pathways (5/15 pathways) of profile 18 or profile 19. Then, the 50 important enzymes in total obtained from these 15 key pathways of profile 18 and profile 19 constructed the protein-protein interaction (PPI) network by the Search Tool for the Retrieval of Interacting Genes (STRING) (Fig. 5). Based on the connectivity degree of each node, 25 key enzymes counted high connectivity degree of each node were finally identified: gelsolin (GSN), YES proto-oncogene 1 (YES1), cytokine inducible SH2-containing protein (CISH), ras homolog family member A (RHOA), protein kinase C, epsilon (PRKCE), Raf-1 proto-oncogene, serine/threonine kinase (RAF1), BCL2 associated agonist of cell death (BAD), suppressor of cytokine signaling 3 (SOCS3), phosphatidylinositol-4,5-bisphosphate 3-kinase catalytic subunit alpha (PIK3CA), v-akt murine thymoma viral oncogene homolog 1 (AKT1), kinase insert domain receptor (KDR), epidermal growth factor receptor (EGFR), tumor protein p53 (TP53), jun proto-oncogene (JUN), SMAD family member 4 (SMAD4), 3-keto-acyl-CoA thiolase 2 (PKT2), mitogen-activated protein kinase 14 (MAPK14), growth factor receptor bound protein 2 (GRB2), vascular endothelial growth factor A (VEGFA), v-myc avian myelocytomatosis viral oncogene homolog (MYC), tumor necrosis factor (TNF), dishevelled segment polarity protein 2 (DVL2), ROS proto-oncogene 1, receptor tyrosine kinase (ROS1), ets variant 5 (ETV5) and frizzled class receptor 10 (FZD10). Key DEGs encoding these 25 enzymes need further studies to clarify the associations between genes and the formation of intestinal air-breathing function in the loach.

### Validation of RNA-Seq results by quantitative real-time RT-PCR (qRT-PCR)

To validate the RNA-seq data obtained in the current study, qRT-PCR of 9 randomly selected DEGs were performed, including intraflagellar transport protein 22 homolog (*IFT22*), serine/threonine-protein phosphatase 2A catalytic subunit beta isoform (*PP2AB*), *VEGFAa*, glutamate dehydrogenase (*gdh*), annexin A1a (*anxa1a*), vasoactive intestinal peptide (*VIP*), metallothionein (*Mt*), protein phosphatase 1 (*PP1*), and spondin 1b (*Spon1b*). Results obtained by qRT-PCR were compared to data obtained by RNA-seq (Supplementary Fig. S6). The expression patterns for the 9 DEGs obtained using the two methods were similar, confirming the reliability of the data obtained by RNA-seq.

## Discussions

The acquisition of atmospheric O_2_ shows important difference from water breathing. *M. anguillicaudatus* can exchange gases both with water, *via* gills and skin, and with air, *via* the posterior region of the intestine^1^. The first two types are common to other fish and amphibian. Intestinal respiration, however, is found in Cobitidae and because of this accessory type of respiration, *M. anguillicaudatus* is adapted to the low oxygen tensions (hypoxia) common in its habitat^4^. Besides, *M. anguillicaudatus* is widely distributed in China, Korea, Japan, and other countries in southeastern Asia^2^. Therefore, the loach can be used as an excellent animal for exploration the formation mechanisms of intestinal air-breathing function. Structures of the loach intestine in relation to respiration have been studied many times^3^. The posterior intestine with a thin muscular wall is rich in erythrocytes and blood vessels, while the anterior intestine possesses a thick muscular wall and abundant mucosal folds^4^, which was consistent with histological observations of the anterior intestine and posterior intestine in this study. And more importantly, in this study, 10-Dph loach showed numerous air bubbles in the intestinal lumen, suggesting the initial formation period of intestinal air-breathing function of loach began at around 10 days post hatch. All the time, scientists have been attracted by structures of the posterior intestine, respiratory zone of loach^1^. Very litter is known on the development and molecular aspects of the intestinal air-breathing function. The expansion of sequencing technologies has sped up the generation of transcriptomics data even for scarce species^6^. Here, high-throughput sequencing of posterior intestine mRNAs at different development stages, including 4-Dph, 8-Dph (before the formation of intestinal air-breathing function) and 12-Dph, 20-Dph, 40-Dph and Oyd (with intestinal air-breathing function) were used to identify genes involved in formation of intestinal air-breathing function in the loach.

As expected, the significantly enriched GO terms that were identified in the present study were mainly associated with metabolic process and molecular function including binding, catalytic activity and substance transport, which was similar to the findings for gene expression profile in the intestine of sea cucumber^10^. As we known, transition from larval to juvenile stage in fish involves development of most organs and tissues, and maturation of different physiological functions^11^. Therefore, GO enrichment analysis of DEGs during the posterior intestine developmental process (4-Dph group, 8-Dph group, 12-Dph group, 20-Dph group, 40- Dph group and Oyd group) of loach was complicated. DEGs obtained here mapped to the enriched GO term cellular process, single-organism process, biological regulation, developmental process and multicellular organismal process, which was also found in European seabass during its early development^12^. These suggested gene regulation was crucial for achieving various physiological functions associated to the development of loach posterior intestine, which was similar to the intestine regeneration process in sea cucumbers since they endured both proliferation and development^13^.

Oxidative phosphorylation^14^ can produce energy for the whole development of loach posterior intestine. Wnt signaling is a highly conserved cellular communication system that is involved in a wide variety of developmental and adult processes in all metazoan organisms^15^. Its mediated effects are diverse, ranging from proliferation, apoptosis, migration, polarization, to stem cell maintenance and differentiation^16^. Notch genes encode transmembrane receptors that are highly conserved from invertebrates to mammals^17^. Notch-mediated signals regulate cell fate decisions in a large number of developmental systems^18^. Hedgehog (Hh) proteins are fundamental to animal development and are conserved in species ranging from Drosophila melanogaster to humans^19^. The mammalian family of Hh proteins includes sonic hedgehog (Shh), desert hedgehog (Dhh), and indian hedgehog (Ihh). These ligands signal through a mechanism involving two transmembrane proteins: patched homolog 1 (Ptch1) and smoothened (Smo)^20^. Mitogen-activated protein kinases (MAPKs) are serine-threonine protein kinases that play an important role in the regulation of many cellular processes including cell growth and proliferation, differentiation, and apoptosis. MAPKs consist of growth factor-regulated extracellular signal-related kinases (ERKs), and the stress-activated MAPKs, c-jun NH2-terminal kinases (JNKs) and p38 MAPKs^21^. Genes encoding components of TGF-β signaling pathway, including ligands TGF-β1 and TGF-β2 as well as receptor TGFBRI, are functionally polymorphic in humans^22^. TGF-β can regulate such diverse processes as cell proliferation, differentiation, motility, adhesion, organization, and programmed cell death^17^. Wnt signaling pathway, Notch signaling pathway, Hedgehog signaling pathway, MAPK signaling pathway and TGF-β signaling pathway are also involved in vasculogenesis and angiogenesis^17^,^21^. Besides, the VEGF pathway is recognized as the key regulators of the angiogenic process^23^. Previous studies have also presented vascular smooth muscle contraction signaling pathway^24^, JAK/STAT1 signaling pathway^25^, insulin signaling pathway^26^ and ErbB signaling pathway^27^ played important roles in angiogenesis. To adapt to the development of loach posterior intestine, such as cell size growing, cell proliferation and migration, the pathways associated with cytoskeleton were found. Regulation of actin cytoskeleton signing pathway^28^, ECM-receptor interaction^29^, osteoclast differentiation^30^ and cell cycle^31^ are mostly associated with the process of cell functions, cell proliferation, differentiation and migration.

To further identify key DEGs which were related to the occurred and formed process of intestinal air-breathing function in loach, the STRING was used for constructing PPI network. Finally, 25 genes were identified: *GSN*^32^, *YES1* 33, *CISH*^34^, *RHOA*^35^, *PRKCE*^36^, *RAF1* 37, *BAD*^38^, *SOCS3* 39, *PIK3CA*^40^, *AKT1* 41, *EGFR*^40^, *TP53* 42, *JUN*^43^, *SMAD4* 44, *PKT2* 45, *MAPK14* 46, *GRB2* 47, *VEGFA*^48^, *MYC*^49^, *TNF*^34^, *DVL2*^50^, *ROS1* 51, *ETV5* 52, *FZD10* 53. These 25 genes were seldom mentioned in the intestinal transcriptomes of the fishes without intestinal air-breathing function, such as zebrafish^9^, European sea bass^54^ and tilapia^8^, while these intestinal transcriptomes mainly told the roles of intestine in nutrition, immune and osmoregulatory functions. In this study, these 25 genes which were mainly involved in development, angiogenesis and cytoskeleton, were thus considered to be related to the intestinal air-breathing function occurred and formed process during posterior intestine development of loach. Further functional characterization of these genes determined by using transgenic, over expression, knockout and knockdown strategies may help elaborate the molecular mechanisms for intestinal air-breathing function formation in the loach.

In conclusion, this report represents the first application of Illumina sequencing technology for transcriptome studies in different developmental stages (4-Dph group, 8-Dph group, 12-Dph group, 20-Dph group, 40- Dph group and Oyd group) of loach posterior intestine, a typical accessory air-breathing organ. A total of 81300 unigenes were generated, and KEGG pathway annotation enabled us to assign 35291 DEGs to 238 pathways. Based on this transcriptome database, we further explored the gene expression profiles in loach posterior intestine, and identified 15 key pathways and 25 key DEGs, thus providing potential targets for candidate gene selection involved in formation of intestinal air-breathing function in *M. anguillicaudatus*. Analysis gained by this study will be useful for future studies on formation of the intestinal air-breathing function in loach. In addition, we demonstrated that *M. anguillicaudatus* is a good model for studies to identify and characterize the molecular basis of accessory air-breathing organ development in fish.

## Methods

### Ethics

This study was conducted in strict accordance with the recommendations in the Guide for the Care and Use of Laboratory Animals of Huazhong Agricultural University. All experimental protocols were approved by the ethics committee of College of Fisheries, Huazhong Agricultural University. All efforts were made to minimize suffering of the loaches.

### Preparation of tissues and histological observation

All the diploid loaches were obtained from our laboratory breeding populations, which were raised in the Aquaculture Base of College of Fisheries, Huazhong Agricultural University in Wuhan City, Hubei Province, China. Firstly, the posterior intestines from the one-year-old diploid loaches were used for RNA extraction and histological observation, meanwhile, the anterior intestines from them were used only for histological observation. Following, mature, healthy and two-year-old diploid loaches were served for reproduction. The pre-experiment showed that the loach would start to swallow air around 10-Dph, suggesting the initial formation period of intestinal air-breathing function of loach began at around 10 days post hatch. Therefore, offspring of two year old diploid loaches were collected at the following developmental stages for histological observation, including 4-Dph, 6-Dph, 8-Dph, 10-Dph, 12-Dph, 20-Dph and 40-Dph. With combination of histological observation of the posterior intestine and loach behavior observation, the different developmental stages (Table 3) selected for developmental transcriptome analysis and identification of genes involved in formation of intestinal air-breathing function, finally included 4-Dph (none), 8-Dph (before the occurrence of intestinal air-breathing), 12-Dph (early stage of the formation of intestinal air-breathing), 20-Dph (mature stage of the formation of intestinal air-breathing), 40-Dph (stable stage of the formation of intestinal air-breathing) and Oyd (stable stage of the formation of intestinal air-breathing). After anesthetizing, intestines of loaches from 4-Dph and posterior intestines of loaches from 8-Dph, 12-Dph, 20-Dph were dissected by needles under a dissecting microscope (Zeiss Stemi 2000-C), while posterior intestines of loaches from 40-Dph were dissected by scissors and tweezers. All intestine tissues (4-Dph, 8-Dph, 12-Dph, 20-Dph, 40-Dph and Oyd) were harvested, quickly frozen in liquid nitrogen and stored at −80 °C prior to RNA extraction. The experimental loaches were cultured in a flowing water with 6.5 ± 0.7 mg.L^−1^ dissolved oxygen and 25.0 ± 2 °C water temperature. Loaches during the larval and early juvenile stages were fed four times a day, and adult loaches were fed twice a day. All the samples used for histological observation were obtained and preserved in 4% paraformaldehyde at room temperature. After dehydration in a graded series of ethanol and transparency by xylene, the posterior intestine samples were embedded in paraffin and sectioned (5 μm thick), using a Leica RM 2135 rotary microtome (Leica Ltd, Wetzlar, Germany). Dewaxed serial sections were stained with HE staining. Images were taken using a Zeiss Axiovert imaging system.

### RNA extraction, RNA-seq library construction and sequencing

Total RNA was extracted from approximately 15 individuals per developmental stage using Trizol (Invitrogen, CA, USA) according to the manufacturer’s instructions. Then the total RNA was treated with RNase-free DNase I (Takara Bio, Japan) for 30 min at 37 °C to remove residual DNA. NanoDrop 2000 (Thermo Scientific, Wilmington, DE, USA) and RNase free agarose gel electrophoresis were performed to assess the quantity and quality of total RNA. The total RNA extracted from specimens of each development stage was pooled together as one stage-specific sample. Poly (A) mRNA was isolated using oligo-dT beads (Qiagen). All mRNA was broken into short fragments by adding fragmentation buffer. First-strand cDNA was generated using random hexamer-primed reverse transcription, followed by the synthesis of the second-strand cDNA using RNase H and DNA polymerase I. The cDNA fragments were purified using a QIAquick PCR extraction kit. These purified fragments were then washed with EB buffer for end reparation poly (A) addition and ligated to sequencing adapters. Following agarose gel electrophoresis and extraction of cDNA from gels, the cDNA fragments were purified and enriched by PCR to construct the final cDNA library. The cDNA library sequencing was carried out with Illiumina HiSeq 2500 RNA-Seq (Illumina, San Diego, CA, USA) using the paired-end technology by Gene Denovo Company (Guangzhou, China).

### *De novo* assembly and functional annotation

The high-quality sequences were required for *de novo* assembly analysis. Prior to assembly and mapping, a filter was used to remove low-quality reads, which were defined as those with adaptor or ambiguous bases (‘N’ < 5%), as well as all reads with nucleotides that had Phred quality scores <20, where a Phred score of 20 corresponds to a 1% expected error rate. Then all clean reads of the six libraries were jointly assembled into unigenes employed by Trinity software. Since there is no reference genome of *M. anguillicaudatus*, a k-mer value cutoff of 25 was used after removing redundant nucleotide sequences by Tgicl (v2.1, http://sourceforge.net/projects/tgicl/files/tgicl%20v2.1/).

Finally, unigenes were aligned against the Nr protein database, Swissprot, KOG, and KEGG of protein database using BlastX with an E-value < 10^−5^. The sequence direction of the unigenes was determined according to the best alignment results. When the results were conflicted among databases, the direction was determined consecutively by Nr, Swissprot, KOG and KEGG. When a unigene would not align to any database, ESTScan (http://myhits.isb-sib.ch/cgi-bin/estscan) was used to predict coding regions and determine sequence direction. GO annotation was analyzed by Blast2GO software (https://www.blast2go.com/). Functional classification of the unigenes was performed using WEGO software.

### Enrichment, dynamic expression profile and protein-protein interaction (PPI) network analysis of differentially expressed genes (DEGs)

The mapped fragments were normalized for RNA length according to fragment per kilobase of exon model per million mapped reads (FPKM) for each gene between the six pooled samples, which facilitated the comparison of transcript levels between samples. DEGs between the two libraries (4-Dph_vs_8-Dph, 8-Dph_vs_12-Dph, 12-Dph_vs_20-Dph, 20-Dph_vs_40-Dph, 40-Dph_vs_ Oyd, 4-Dph_vs_12-Dph, 4-Dph_vs_20-Dph, 4-Dph_vs_40-Dph and 4-Dph_vs_ Oyd) were identified by the DEGseq package using the MA-plot-based method with Random Sampling model (MARS) method^55^. DEGs between the each two libraries were selected using the following filter criteria: FDR (False discovery rate) <0.01 and the absolute value of log_2_Ratio ≥1, meaning that the expression difference for each DEG in different libraries should be at least two-fold. Go enrichment analysis (p-value ≤ 0.05) of the DEGs was performed using GOseq with the Wallenius non-central hyper-geometric distribution model to adjust gene length bias in DEGs. KEGG pathway enrichment analysis of the DEGs was done using KOBAS^56^ with the hyper-geometric distribution model. The enrichment p-values were adjusted using the Benjamin and Hochberg method. In addition, the determination of expression patterns of DEGs through developmental stages was completed with clusters generated by STEM^57^.

The interaction relationships of the enzymes encoded by DEGs that were obtained from STRING database (http://string-db.org/) providing both experimental and predicted interaction information. This database was used to analyze PPI network for DEGs by calculating their required confidence score; a score > 0.4 was chosen as the cut-off criterion.

### Validation of RNA-Seq Results by qRT-PCR

To examine the reliability of the RNA-Seq results, 9 differently expressed genes were selected randomly for validation by qRT-PCR: *IFT22*, *PP2AB*, *VEGFAa*, *gdh*, *anxa1a*, *VIP*, *Mt*, *PP1* and *Spon1b*. Total RNA was extracted from *M. anguillicaudatus* at six developmental stages as described above. The cDNA was synthesized from 3 μg of total RNA for each sample using Prime-ScriptTM RT reagent Kit (TaKaRa, Dalian, China). Primers (shown in Supplementary Table S4) were designed using Primer Premier 5.0. Reactions were performed with SYBR^®^ Premix Ex Taq™ kit (TaKaRa) in Bio-Rad CFX96 Real-Time PCR system. The PCR mixture consisted of 12.5 μl SYBR Premix Ex Taq II, 1 μl template cDNA, 0.5 μl forward primer (10 μM), 0.5 μl reverse primer (10 μM) and 10.5 μl ddH_2_O. qRT-PCR detection system was: denature at 95 °C for 30 s, followed by 39 cycles of 5 s at 95 °C, and 58 °C for 30 s. Following the amplification, a dissociation curve was performed to verify that a single product was generated. Each sample was run in four times technical replicates. The relative expression levels were normalized to the endogenous control gene *β-actin*, and expression ratios were calculated by using the 2^−ΔΔCt^ method.

## Additional Information

**Accession codes**: All transcriptome data are available in the NCBI Short Read Archive (SRA) database under accession SRP072927.

**How to cite this article**: Luo, W. *et al.* Developmental transcriptome analysis and identification of genes involved in formation of intestinal air-breathing function of Dojo loach, *Misgurnus anguillicaudatus. Sci. Rep.* **6**, 31845; doi: 10.1038/srep31845 (2016).

## Supplementary Material

Supplementary Information

## Figures and Tables

**Table 1:** Overview of the sequencing reads.

Samples	4-Dph	8-Dph	12-Dph	20-Dph	40-Dph	Oyd
Raw read number	38347890	40177226	36590380	37786998	32886306	58152842
Clean read number	37787284	39660682	36031650	37275524	32448448	48367330
Raw read base pairs	4793486250	5022153250	4573797500	4723374750	4110788250	5873437042
Clean read base pairs	4723410500	4957585250	4503956250	4659440500	4056056000	5136684000
Q20%	97.41%	97.67%	97.50%	97.50%	97.67%	97.74%
Q30%	94.64%	95.14%	94.83%	94.81%	95.18%	91.79%
GC%	48.42%	48.33%	47.43%	47.22%	46.58%	46.98%

Q20: The percentage of bases with quality value larger than 20.

Q30: The percentage of bases with quality value larger than 30.

GC%: The percentage of proportion of guanidine and cytosine nucleotides among total nucleotides.

**Table 2:** Key pathways associated with the formation of intestinal air-breathing function of *M. anguillicaudatus.*

#Pathway	Pathway ID
Regulation of actin cytoskeleton	Ko04810
MAPK signaling pathway	Ko04010
VEGF signaling pathway	Ko04370
Notch signaling pathway	Ko04330
Vascular smooth muscle contraction	Ko04270
Wnt signaling pathway	Ko04310
Insulin signaling pathway	Ko04910
ECM-receptor interaction	Ko04512
Jak-STAT signaling pathway	Ko04630
TGF-beta signaling pathway	Ko04350
ErbB signaling pathway	Ko04012
Oxidative phosphorylation	Ko00190
Hedgehog signaling pathway	Ko04340
Osteoclast differentiation	Ko04380
Cell cycle	Ko04110

**Table 3:** Characteristics of *M. anguillicaudatus* during intestinal air-breathing function occurred and formed process.

Sample names	Histological characteristics of the posterior intestines	Developmental stage of intestinal air-breathing
4-Dph	The intestinal epithelium was simple columnar epithelium.	None (No intestinal air-breathing phenomenon was observed)
8-Dph	The epithelium of posterior intestine was still simple columnar epithelium.	Before the occurrence of intestinal air-breathing (No intestinal air-breathing phenomenon was observed)
12-Dph	A certain amount of erythrocytes were found in posterior intestine and the loach swam near the surface to swallow the air.	Early stage of the formation of intestinal air-breathing (The intestinal air-breathing phenomenon was observed)
20-Dph	The number of erythrocytes and blood capillaries increased in posterior intestine.	Mature stage of the formation of intestinal air-breathing (The intestinal air-breathing function obviously enhanced and matured)
40-Dph	The posterior intestine was abundant in erythrocytes and blood capillaries.	Stable stage of the formation of intestinal air-breathing
Oyd	The posterior intestine was abundant in erythrocytes and blood capillaries.	Stable stage of the formation of intestinal air-breathing
