# Supplementary material for: Developmental transcriptome analysis and identification of genes involved in formation of intestinal air-breathing function of Dojo loach, Misgurnus anguillicaudatus
Source: Sci Rep. 2016 Aug 22;6:31845. doi: 10.1038/srep31845 (PMC4992823; doi:10.1038/srep31845)
Supplement: Supplementary Information [file srep31845-s1.doc]

**Developmental transcriptome analysis and identification of genes involved in formation of intestinal air-breathing function of Dojo loach, *Misgurnus anguillicaudatus***

Weiwei Luo^1^, Xiaojuan Cao^1,2*^, Xiuwen Xu^1^, Songqian Huang^1^, Chuanshu Liu^1^, Tea Tomljanovic^3^

^1^College of Fisheries, Key Lab of Agricultural Animal Genetics, Breeding and Reproduction of Ministry of Education/Key Lab of Freshwater Animal Breeding, Ministry of Agriculture, Huazhong Agricultural University, Wuhan 437000, Hubei, People’s Republic of China

^2^Freshwater Aquaculture Collaborative Innovation Center of Hubei Province, Hubei, People’s Republic of China

^3^Department for Fisheries, Beekeeping, Game management and Special Zoology, Faculty of Agriculture, University of Zagreb, Zagreb, Croatia

**E-mail address for all authors:**

Luo WW: [weiweiluo66@163.com](mailto:weiweiluo66@163.com),

Cao XJ: [caoxiaojuan@mail.hzau.edu.cn](mailto:caoxiaojuan@mail.hzau.edu.cn), *Corresponding author

Xu XW: xiuwenxu1207@163.com ,

Huang SQ: [huangsongqian@163.com](mailto:huangsongqian@163.com),

Liu CS: [chuanshuliu@163.com](mailto:chuanshuliu@163.com),

Tomljanovic T: [ttomljanovic@agr.hr](mailto:ttomljanovic@agr.hr).

**Supplementary Tables and Figures**

**Supplementary Table S1** Summary of the assembly

| Unigene number | GC (%) | N50 | Max length | Min length | Average length |
| --- | --- | --- | --- | --- | --- |
| 81300 | 43.76% | 1662 | 24035 | 228 | 879.78 |

N50: the length L where 50% of all nucleotides in the assembly are contained in unigenes of size ≥L.

**Supplementary Table S2** Summary of annotations on unigenes against public databases

| Database | Number of annotated unigene | Percent of annotated unigene(%) |
| --- | --- | --- |
| Nr | 40563 | 49.89 |
| Swissprot | 31588 | 38.85 |
| KOG | 24837 | 30.55 |
| KEGG | 17077 | 21.00 |
| Total | 40757 | 50.13 |

**Supplementary Table S3 Number of DEGs at each two consecutive time points**

| Comparisons | Number of up-regulated genes | Number of down-regulated genes | Number of all DEGs |
| --- | --- | --- | --- |
| Dph-4-VS-Dph-8 | 2461 | 7818 | 10279 |
| Dph-8-VS-Dph-12 | 7317 | 2558 | 9875 |
| Dph-12-VS-Dph-20 | 5070 | 2037 | 7107 |
| Dph-20-VS-Dph-40 | 7702 | 3428 | 11130 |
| Dph-40-VS-Oyd | 11383 | 10078 | 21461 |

**Supplementary Table S4 Primer sequences of the DEGs for qRT-PCR**

| Gene | Forward primer(5'-3') | Reverse primer(5'-3') | Length(bp) |
| --- | --- | --- | --- |
| *IFT22* | TGGGATTGTGGAGGAGATTTC | AGTTTGCTCAGTTTTGGGGC | 245 |
| *PP2AB* | ACAGTCACACTTCTTGTTGCCCT | ATTTCCTCAAGCACTCGTCGTA | 124 |
| *VEGFAa* | TCTGCTCTATAACCCTCACCGC | GTCATTTTTGCTCTTCCCTCCT | 153 |
| *gdh* | TGCCTGTGTGACTGGTAAGCC | CCATAACGGTGAAGATAACGCA | 216 |
| *anxa1a* | TGCTGTGGTGAAATGTGCTG | AGTCTCCTTTGGTGTCGTCCT | 209 |
| *VIP* | GTCTCTTCACAAGCGGATACAG | TGGTCCTCCATCAAATCATCAC | 106 |
| *Mt* | GAAACGATACAGCAAAGGAACC | CTTACAAACGCATCCAGAGGC | 204 |
| *PP1* | GAGGACGGTTATGAGTTTTTTGC | GCTTTCTTCTCTGACGGCTTG | 155 |
| *Spon1b* | GTCGGACGGTTTCTGTAGGA | GAGGGTAAATCCACGAAAGTAAG | 172 |

# Gene abbreviations: intraflagellar transport protein 22 homolog (*IFT22*); serine/threonine-protein phosphatase 2A catalytic subunit beta isoform (*PP2AB*); vascular endothelial growth factor Aa (*VEGFAa*); glutamate dehydrogenase (*gdh*); annexin A1a (*anxa1a*); [vasoactive intestinal peptide](http://www.ncbi.nlm.nih.gov/nuccore/NM_001113190.1) (*VIP*); metallothionein (*Mt*); protein phosphatase 1 (*PP1*); [spondin 1b](http://www.ncbi.nlm.nih.gov/nuccore/BC162653.1) (*Spon1b*).


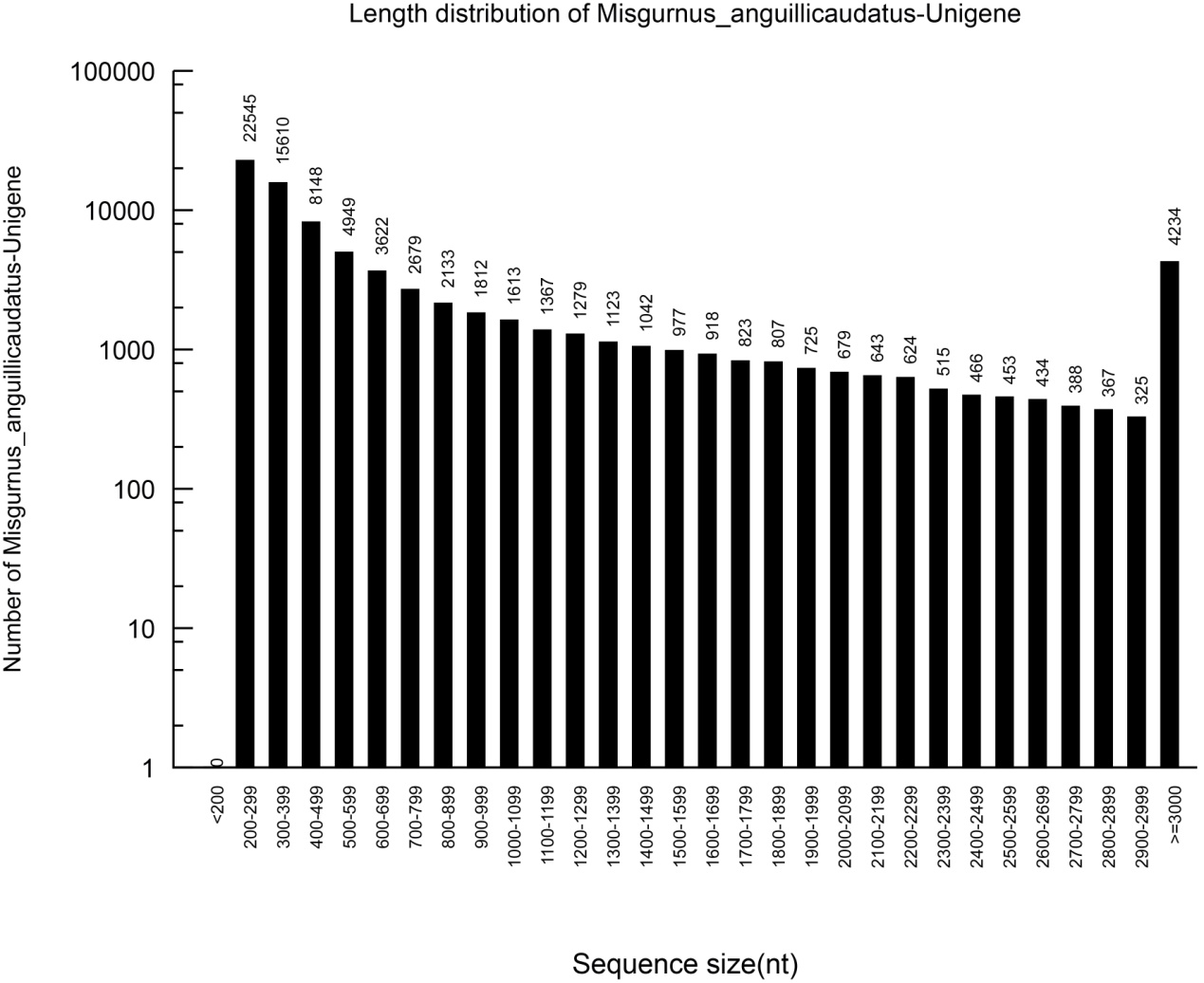


**Supplementary Fig. S1 The size distribution of the unigenes.**


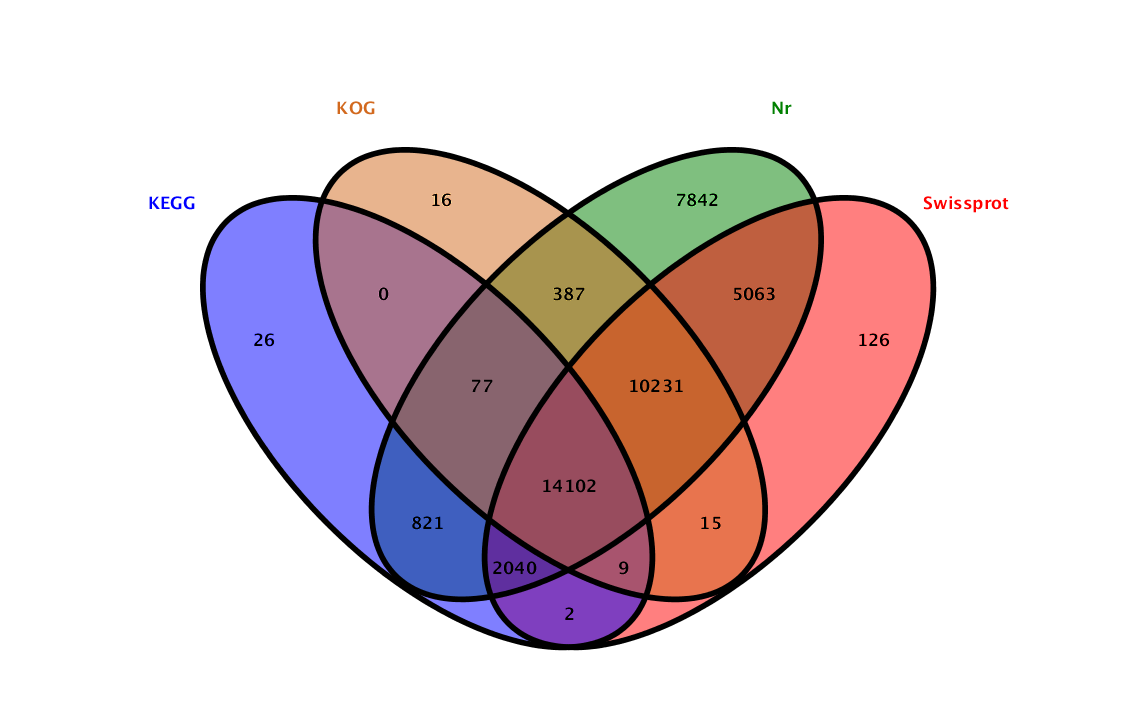


**Supplementary Fig. S2 Detection of homologous genes in public databases.**


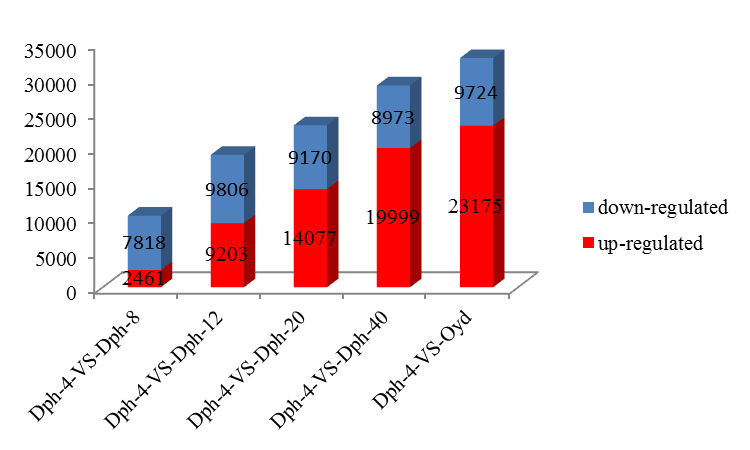


**Supplementary Fig. S3 Number of DEGs in posterior intestine of *M. anguillicaudatus* among different developmental stages.**

**
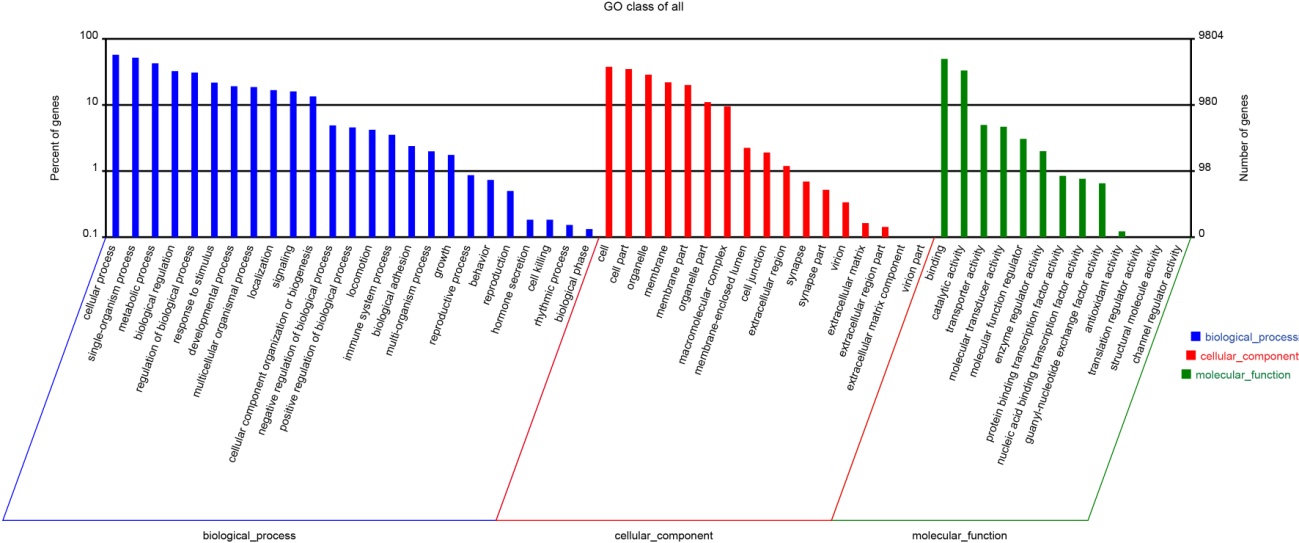
**

**Supplementary Fig. S4 GO classifications of DEGs from posterior intestine of *M. anguillicaudatus*.**


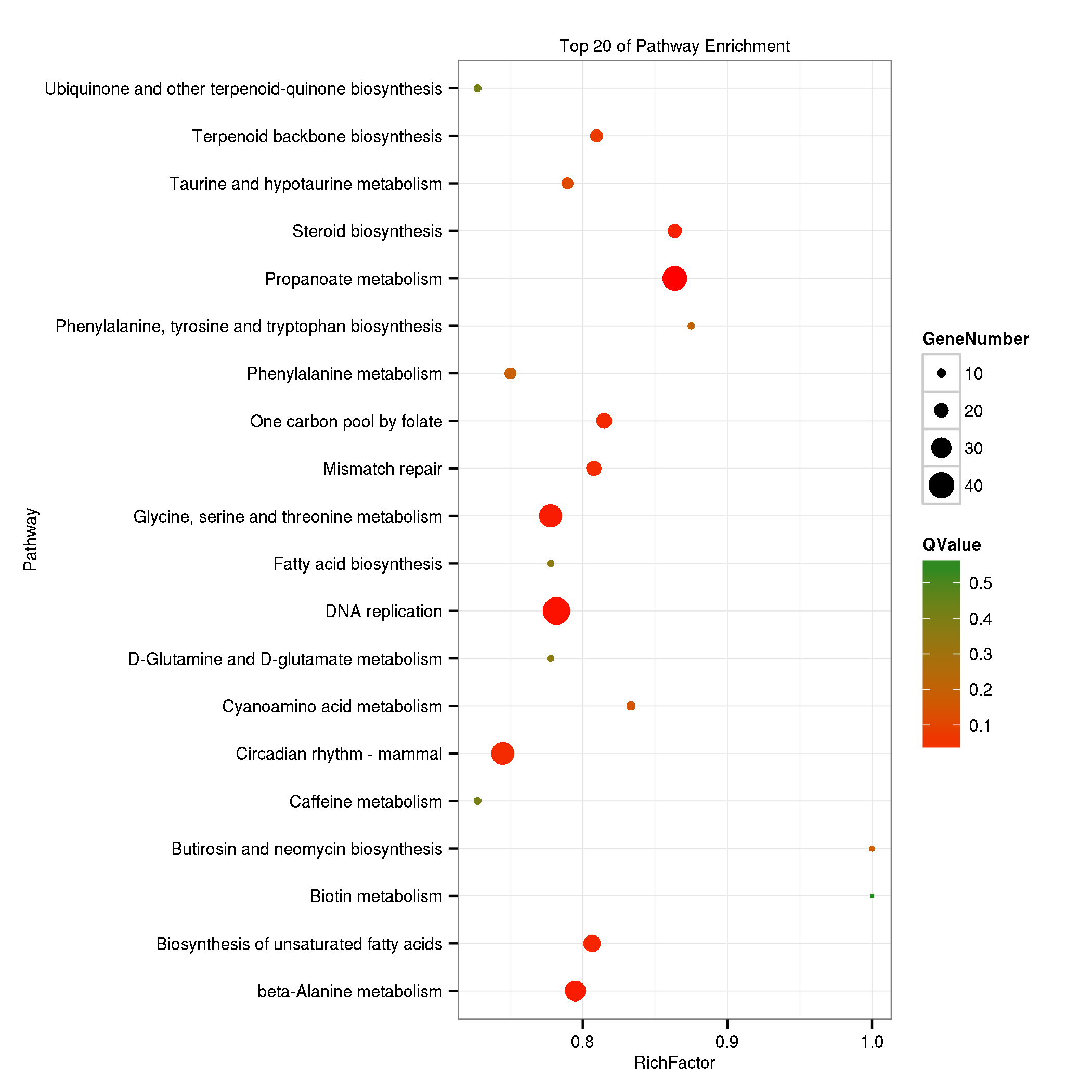


**Supplementary Fig. S5 The top 20 significantly enriched KO pathways of all DEGs.**


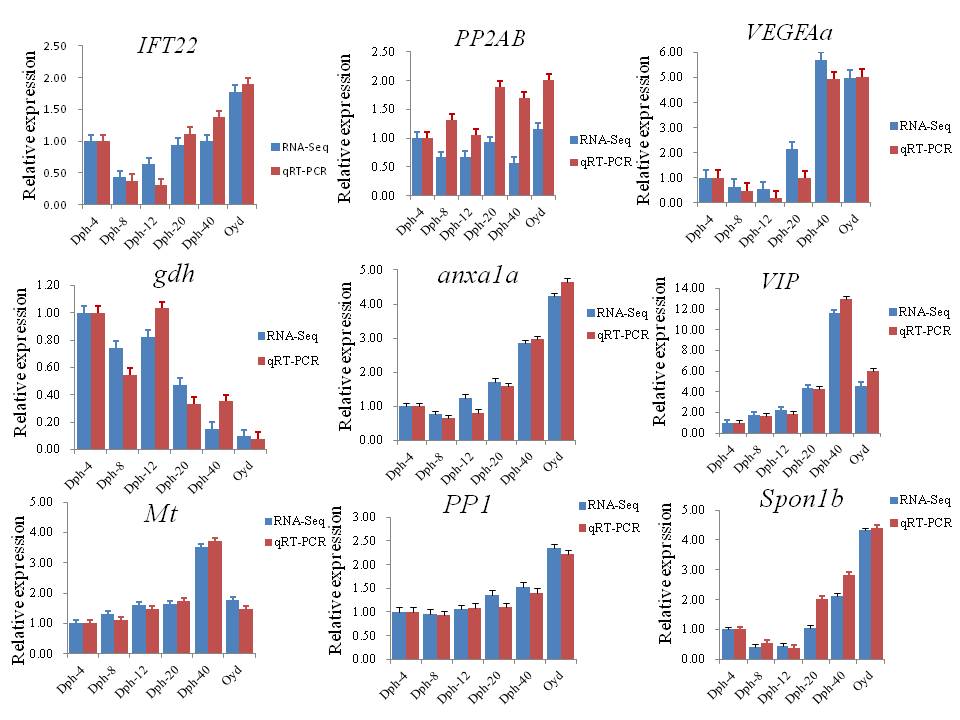


**Supplementary Fig. S6 qRT-PCR validation of differently expressed genes in posterior intestine of *M. anguillicaudatus* during different developmental stages.** Gene expressions were expressed as mean normalized ratios (n=4, ±SE). Gene abbreviations: intraflagellar transport protein 22 homolog (*IFT22*); serine/threonine-protein phosphatase 2A catalytic subunit beta isoform (*PP2AB*); vascular endothelial growth factor Aa (*VEGFAa*); glutamate dehydrogenase (*gdh*); annexin A1a (*anxa1a*); [vasoactive intestinal peptide](http://www.ncbi.nlm.nih.gov/nuccore/NM_001113190.1)(*VIP*); metallothionein (*Mt*); protein phosphatase 1 (*PP1*); [spondin 1b](http://www.ncbi.nlm.nih.gov/nuccore/BC162653.1) (*Spon1b*).
